# Prognostic significance and therapeutic implications of centromere protein F expression in human nasopharyngeal carcinoma

**DOI:** 10.1186/1476-4598-9-237

**Published:** 2010-09-09

**Authors:** Jing-Yan Cao, Li Liu, Shu-Peng Chen, Xing Zhang, Yan-Jun Mi, Zhi-Gang Liu, Man-Zhi Li, Hua Zhang, Chao-Nan Qian, Jian-Yong Shao, Li-Wu Fu, Yun-Fei Xia, Mu-Sheng Zeng

**Affiliations:** 1 State Key Laboratory of Oncology in South China; 2 Department of Experimental Research, Sun Yat-Sen University Cancer Center, Guangzhou, 510060, China; 3 Department of Radiotherapy, Sun Yat-Sen University Cancer Center, Guangzhou, 510060, China; 4 Department of Medical Oncology, The 3rd Affiliated Hospital of Harbin Medical University, Harbin, 150040, China; 5 Department of Nasopharyngeal Carcinoma, Sun Yat-Sen University Cancer Center, Guangzhou, 510060, China; 6 Department of Biotherapy, Sun Yat-Sen University Cancer Center, Guangzhou, 510060, China

## Abstract

**Background:**

Our recent cDNA microarray data showed that centromere protein F (CENP-F) is significantly upregulated in primary cultured nasopharyngeal carcinoma (NPC) tumor cells compared with normal nasopharyngeal epithelial cells. The goal of this study was to further investigate the levels of CENP-F expression in NPC cell lines and tissues to clarify the clinical significance of CENP-F expression in NPC as well as the potential therapeutic implications of CENP-F expression.

**Methods:**

Real-time RT-PCR and western blotting were used to examine CENP-F expression levels in normal primary nasopharyngeal epithelial cells (NPEC), immortalized nasopharyngeal epithelial cells and NPC cell lines. Levels of CENP-F mRNA were determined by real-time RT-PCR in 23 freshly frozen nasopharyngeal biopsy tissues, and CENP-F protein levels were detected by immunohistochemistry in paraffin sections of 202 archival NPC tissues. Statistical analyses were applied to test for prognostic associations. The cytotoxicities of CENP-F potential target chemicals, zoledronic acid (ZOL) and FTI-277 alone, or in combination with cisplatin, in NPC cells were determined by the MTT assay.

**Results:**

The levels of CENP-F mRNA and protein were higher in NPC cell lines than in normal and immortalized NPECs. CENP-F mRNA level was upregulated in nasopharyngeal carcinoma biopsy tissues compared with noncancerous tissues. By immunohistochemical analysis, CENP-F was highly expressed in 98 (48.5%) of 202 NPC tissues. Statistical analysis showed that high expression of CENP-F was positively correlated with T classification (*P *< 0.001), clinical stage (*P *< 0.001), skull-base invasion (*P *< 0.001) and distant metastasis (*P *= 0.012) inversely correlated with the overall survival time in NPC patients. Multivariate analysis showed that CENP-F expression was an independent prognostic indicator for the survival of the patient. Moreover, we found that ZOL or FTI-277 could significantly enhance the chemotherapeutic sensitivity of NPC cell lines (HONE1 and 6-10B) with high CENP-F expression to cisplatin, although ZOL or FTI-277 alone only exhibited a minor inhibitory effect to NPC cells.

**Conclusion:**

Our data suggest that CENP-F protein is a valuable marker of NPC progression, and CENP-F expression is associated with poor overall survival of patients. In addition, our data indicate a potential benefit of combining ZOL or FTI-277 with cisplatin in NPC suggesting that CENP-F expression may have therapeutic implications.

## Background

Nasopharyngeal carcinoma (NPC) is a disease with remarkable geographic and racial distributions worldwide. It is one of the most common cancers in Southeastern Asia and is highly prevalent among populations originating from Southern China where the yearly incidence rate of NPC is 25-50 per 100,000 people [1,2]. In North America and other western countries, the yearly incidence is less than 1 per 100,000 [1]. NPC is a particular type of squamous carcinoma of head and neck associated with EBV infection, environmental factors and genetic aberrance [3]. Most NPCs are undifferentiated or poorly differentiated with the following characteristics: fast growth and a great tendency to invade adjacent regions as well as metastasize to regional lymph nodes and distant organs. Although NPCs are usually radiosensitive, local failure and metastasis still occur [4,5]. Nasopharyngeal carcinogenesis is a multi-step process with morphological progression involving multiple genetic and epigenetic events [6]. Thus, identification of molecular and biological changes that occur during carcinogenesis and progression could facilitate investigation of the pathology of the disease and generate new prognostic markers to more accurately predict patients' clinical outcome, helping to individualize treatments for NPC patients.

CENP-F (or mitosin) is a member of the human centromeric proteins (CENPs) family, which is involved in centromere formation and kinetochore organization during mitosis [7,8]. Its expression and subcellular localization patterns are regulated in a cell cycle-dependent manner. No detectable expression of CENP-F has been reported in G0/G1, only low levels of expression have been detected in the nuclear matrix during S phase, and CENP-F proteins gradually accumulate in the nucleus in G2 then localizing to kinetochores in mitosis and reach the maximal expression in G2 and M cells [9]. At the end of mitosis, CENP-F is rapidly proteolyzed by the proteasome [10]. Accumulating evidence suggests that CENP-F is an important protein involved in chromosome alignment and kinetochore-microtubule interaction. Depletion of CENP-F results in chromosome misalignment and improper microtubule-kinetochore attachment [11]. It interacts directly with many proteins including CENP-E, NudE, ATF4, and Rb, thereby modulating cell fate [12]. The kinetochore-targeting domain is located near the C-terminus, a region that is sensitive to farnesyltransferase inhibitors (FTIs), which can prevent CENP-F farnesylation and cause mitotic chromosome alignment defects [13]. A recent report showed that zoledronic acid (ZOL) can inhibit farnesylation of CENP-F and disrupt proper localization and functioning of the protein [14].

Cell cycle-specific expression of CENP-F makes it a potential marker of proliferation. Indeed, CENP-F is correlated with tumor proliferation in a variety of human tumors, including lung cancer [15], non-Hodgkin lymphoma [16], salivary gland tumors [17], and mantle cell lymphoma [18]. CENP-F is also correlated with early recurrence in intracranial meningiomas [19] and poor prognosis in breast cancer [20]. The CENP-F gene is located on 1q32-q41, which is frequently amplified in NPCs as shown by comparative genomic hybridization analysis [21]. Using a cDNA microarray, we analyzed the global gene expression profile of primary cultured NPC cells and found that CENP-F is significantly upregulated in NPC cells compared with normal nasopharyngeal epithelial cells [22].

Our previous studies raised important questions regarding patterns of CENP-F expression in human NPC tissues, potential correlations with clinicopathologic grade and prognosis, and its potential role in chemotherapy. Here, we found that CENP-F was upregulated in NPC cell lines and tissues. Immunohistochemistry analysis revealed that CENP-F expression was positively correlated with clinicopathologic features and inversely correlated with overall survival. Cox regression analysis identified CENP-F as an independent factor for clinical prognosis. More importantly, we revealed that combining cisplatin with ZOL or FTI could have synergistic effects in NPC cell lines with high CENP-F expression. Taken together, our results suggest that CENP-F could be a potential prognostic biomarker for clinical outcome and a promising indicator for selective therapeutic treatment in NPC.

## Results

### CENP-F expression is upregulated in NPC cell lines

**Figure 1:**
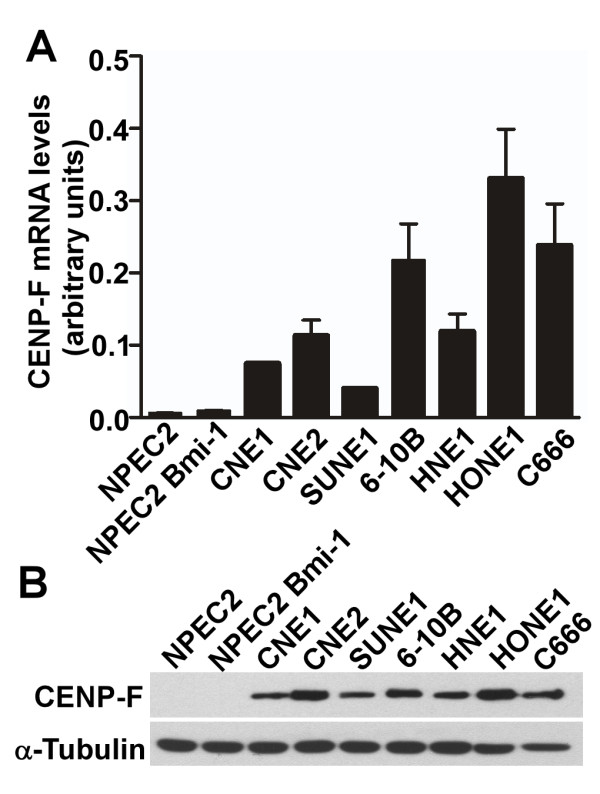
**Expression analysis of CENP-F mRNA and protein in nasopharyngeal epithelial cells**. A. Real-time RT-PCR analysis of CENP-F mRNA in normal nasopharyngeal epithelial cells (NPEC2), immortalized nasopharyngeal epithelial cell line (NPEC2 Bmi-1) and NPC cell lines (CNE1, CNE2, SUNE1, 6-10B, HNE1, HONE1 and C666). B. Western blot analysis of CENP-F protein in the same cell lines as described in A.

To investigate the expression levels of CENP-F, real-time RT-PCR, and western blotting analysis were conducted on the samples of normal nasopharyngeal epithelial cells (NPEC2), the immortalized nasopharyngeal epithelial cells (NPEC2 Bmi-1), and various NPC cell lines. Real-time RT-PCR revealed a higher expression of CENP-F mRNA in all the cancer cell lines than that in NPEC2 and NPEC2 Bmi-1 cells (Fig. 1A). Consistent with mRNA levels, high expression of CENP-F protein was observed in all of the NPC cell lines, whereas there was no detectable expression of CENP-F in NPEC2 and NPEC2 Bmi-1 (Fig. 1B). The high expression of CENP-F was further confirmed in cancer cells after normalized by the percentage of the cells in G2/M (Additional file 1 Fig. S1). Thus, we concluded that all NPC cell lines manifested higher CENP-F expression at both the mRNA and protein levels compared with that of normal and immortalized cells.

### Overexpressions of CENP-F in NPC tissues

**Figure 2:**
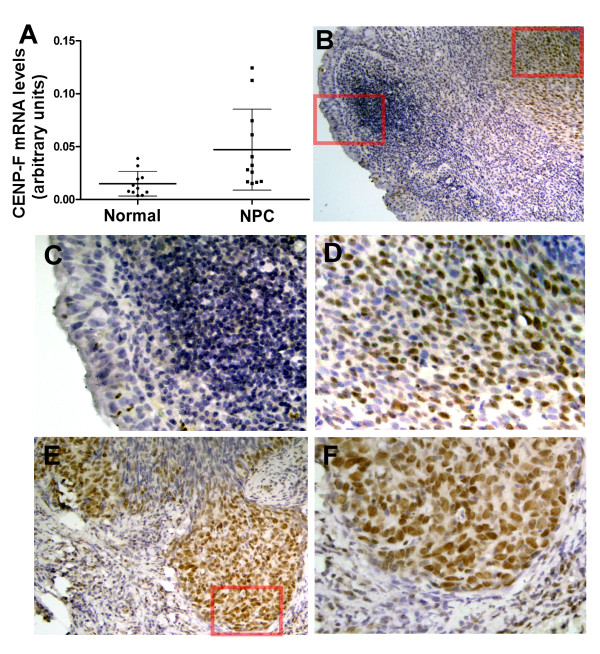
**Expression analysis of CENP-F mRNA and protein in NPC samples**. A. Real-time RT-PCR analysis of CENP-F mRNA in normal nasopharyngeal biopsies (Normal) and nasopharyngeal carcinoma biopsies (NPC). B-F. Immunohistochemistry analysis of CENP-F expression in archived NPC tissue. CENP-F specific expression is observed in tumor cells and not in the uninvolved nasopharyngeal epithelia (B). CENP-F is not expressed in normal epithelial cells (C). CENP-F expression was mainly localized within nuclei of cancer cells (D, E and F), and its expression was observed mainly in the invasive front areas (E and F). B (×100), E (×200) and C, D, F (×400).

To determine the expression of CENP-F in NPC tissues, real-time RT-PCR analysis was performed in 11 noncancerous tissue samples and 12 NPC tissue samples. CENP-F was found to be significantly upregulated in NPC tissue samples compared with noncancerous samples (*P *= 0.0141) (Fig. 2A). To verify this observation, we further examined the expression and localization of CENP-F protein in 202 paraffin-embedded NPC samples by immunohistochemical analysis. Of these archival NPC tissues, 24 contained normal and uninvolved nasopharyngeal columnar epithelia and 12 contained uninvolved nasopharyngeal squamous epithelia. CENP-F protein was detected in 133 of 202 (65.8%) tissues. Strong staining of CENP-F protein was detected in 98 (48.5%) tumors. As shown in Fig. 2, CENP-F specific expression was observed in tumor cells but not in the uninvolved nasopharyngeal epithelia (Fig. 2B). Specific CENP-F was predominantly found to be expressed in carcinoma cells (Fig. 2D-F). However, there was no correlation between the number of CENP-F expressing cells and the number of mitotic cells (Additional file 2 Fig. S2). In contrast, no positive staining or only marginal staining was detected in adjacent noncancerous epithelial cells (Fig. 2C). CENP-F was mainly expressed in the nuclei of tumor cells and preferentially located at the tumor invasive front (Fig. 2E and 2F). These data suggest that CENP-F is overexpressed in NPC samples, especially in the invasive front, which indicates a potential role in promoting tumor invasion.

### Positive correlation of CENP-F expression with clinicopathologic features

Immunohistochemical determination of CENP-F levels was statistically analyzed to identify an association with the clinicopathologic features of NPC. As shown in Table 1, CENP-F expression was significantly correlated with T classification (*P *< 0.001), clinical stage (92 stage) (*P *< 0.001), skull-base invasion (*P *< 0.001), and distant metastasis (*P *= 0.012). However, there was no significant correlation between CENP-F expression and age, gender, histological classification, or N classification. Spearman correlation analysis showed CENP-F expression levels were positively correlated with clinical stage (*r *= 0.395; *P *< 0.001), T classification (*r *= 0.359; *P *< 0.001), skull-base invasion (*r *= 0.245; *P *< 0.001), and distant metastasis (*r *= 0.177; *P *= 0.012) (Table 2). Our data indicates that high CENP-F expression significantly correlated with advanced tumor stage, local invasion, and clinical progress.

**Table 1:** Correlation between the clinicopathologic features and expression of CENP-F protein

Characteristics	CENP-F (%)		*P*
	Low expression	High expression	
**Gender**			
Male	73 (48.3)	78 (51.7)	
Female	31 (60.7)	20 (39.3)	0.124
**Age (y)**			
≤ 45	49 (54.4)	41(45.6)	
> 45	55 (49.1)	57 (50.9)	0.451
**Histologic classification**			
Type II	4 (40.0)	6 (60.0)	
Type III	100 (52.1)	92 (47.9)	0.528*
**Clinical stage (92 stage)**			
I-II	65 (75.6)	21 (24.4)	
III-IV	39(33.6)	77 (66.4)	< 0.001
**T classification**			
T1-T2	82 (66.1)	42 (33.9)	
T3-T4	22 (28.2)	56 (71.8)	< 0.001
**N classification**			
N0	65 (55.1)	53 (44.9)	
N1-3	39 (46.4)	45 (53.6)	0.225
**Distant metastases**			
No	97 (54.8)	80(45.2)	
Yes	7 (28.0)	18 (72.0)	0.012
**Skull-base invasion**			
No	92 (57.9)	67 (42.1)	
Yes	12(27.9)	31 (72.1)	< 0.001

*. Fisher's exact test.

**Table 2:** Spearman correlation analysis between CENP-F and clinicopathologic factors

Variables	CENP-F expression level	
	Spearman correlation	*P*-value
Clinical stage	0.395	< 0.001
T classification	0. 359	< 0.001
Skull-base invasion	0.245	< 0.001
Distant metastases	0.177	0.012

### Inverse correlation of CENP-F expression with patients' survival

**Figure 3:**
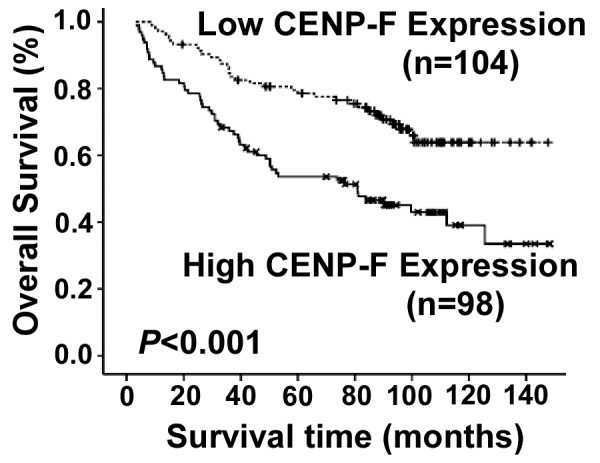
**Kaplan-Meier curves with univariate analyses (log-rank) for patients with low CENP-F expression (dotted line) versus high CENP-F expressing (bold line) groups**. The 5-year overall survival rates were 78.6% in the low CENP-F expression group (*n *= 104), whereas the 5-year overall survival rates were 53.6% in the high expression group (*n *= 98).

We next evaluated whether the level of CENP-F expression was associated with patient prognosis. The median follow-up time for the 202 cases was 85 months with a range from 3 to 148 months. As shown in Fig. 3, the survival time was significantly different between the groups (*P *< 0.001); the low CENP-F expression group had a longer overall survival time, whereas the high CENP-F expression group had a shorter overall survival time. The cumulative 5-year survival rate was 78.6% (95% confidence interval, 0.708-0.864) in the low CENP-F group, but it was only 53.6% (95% confidence interval, 0.436-0.636) in the high CENP-F group.

Besides CENP-F expression level, gender, T classification, N classification, distant metastasis, clinical stage (92 stage), and skull-base invasion were also significantly correlated with survival in Kaplan-Meier analysis and Log-rank test (for gender, *P *= 0.036; for T classification, N classification, distance metastasis, clinical stage, and skull-base invasion, *P *< 0.001). Univariate analysis showed that gender, clinical stage, skull-base invasion and CENP-F expression were statistically significant prognostic factors. However, clinical prognosis was not associated with age or histological classification. Multivariate analysis including CENP-F expression level, gender, clinical stage, and skull-base invasion demonstrated that CENP-F expression level (*P *= 0.045) and clinical stage (*P *< 0.001) were independent prognostic factors for NPC, whereas gender and skull-base invasion were not independent predictors (Table 3). Thus, our findings indicate that CENP-F expression level, as an independent prognostic factor, is inversely associated with clinical prognosis of NPC.

**Table 3:** Univariate and multivariate analysis of different prognostic variables in patients with NPC by Cox regression analysis

	Univariate analysis		Multivariate analysis	
	*P*	HR (95% CI)	*P*	HR (95% CI)
Gender				
Female vs. Male	0.039	0.557 (0.319-0.971)	0.190	0.686 (0.390-1.205)
Clinical Stage				
III-IV vs. I-II	< 0.001	3.188 (1.964-5.175)	< 0.001	2.660 (1.588-4.457)
Skull-base invasion				
Yes vs. No	< 0.001	2.344 (1.482-3.706)	0.229	1.355 (0.825-2.225)
Expression of CENP-F				
High vs. Low	0.001	2.269 (1.472-3.498)	0.045	1.604 (1.011-2.543)

### ZOL Synergizes with cisplatin to enhance the cytotoxicity on CENP-F high expressing cells

**Figure 4:**
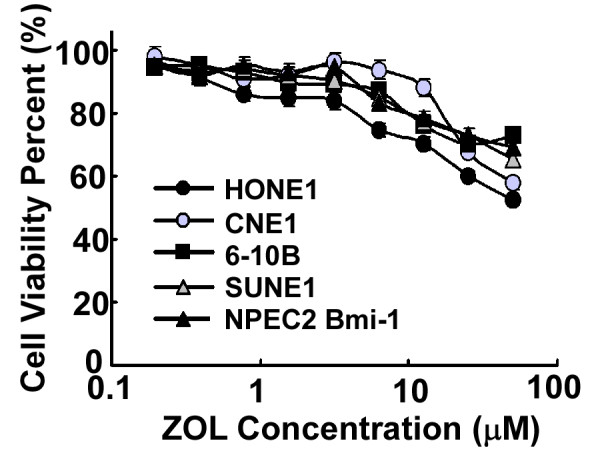
**Dose-response curves for ZOL**. The viability of each cell line (NPEC2 Bmi-1 cell line (black triangle), CNE1 cell line (gray circle), SUNE1 cell line (gray triangle), HONE1 cell line (black circle), 6-10B cell line (black square)) was plotted against the concentration of drug used in a 72 h treatment. Each data point represents the geometric mean of at least three independent experiments.

Recently, CENP-F was suggested to be a potential new molecular target contributing to the anti-tumor effects of ZOL in breast cancer cells [14]. As overexpression of CENP-F was commonly observed in NPC cell lines and tissues, we then analyzed whether NPC cell lines were sensitive to ZOL treatment. As shown by the dose-response curves in Fig. 4, treatment with ZOL alone only exhibited a minor inhibitory effect, and the survival rate of all five cell lines was still more than 60% even at high doses (50 μM). The results indicate that ZOL alone has little cytotoxicity and cannot inhibit proliferation in either immortalized nasopharyngeal epithelial cells with low expression of CENP-F (NPEC2 Bmi-1) or NPC cell lines with elevated expression of CENP-F.

**Figure 5:**
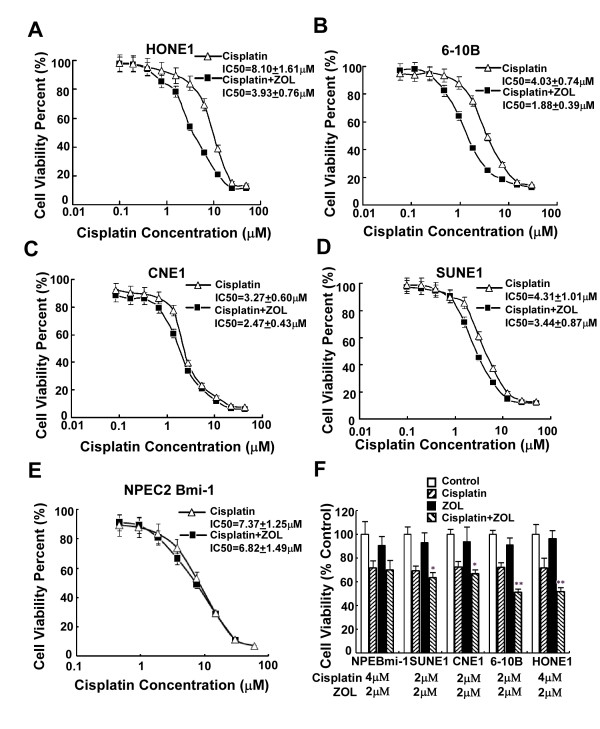
**The cytotoxic effect of cisplatin alone or in combination with ZOL**. A-E. Shifting of dose-response curves of cisplatin by ZOL. Five nasopharyngeal epithelial cells (HONE1(A), 6-10B (B), CNE1 (C), SUNE1 (D) and NPEC2 Bmi-1 (E)) were assayed using MTT assay. The dose-response curves of cisplatin in the presence of ZOL (black square) or the absence of ZOL (triangle). The IC50 values are shown. F. ZOL potentiated effect of cisplatin in all five nasopharyngeal epithelial cells. The concentrations of the drugs used are shown below the names of the cell lines, NPEBmi-1 represents NPEC2 Bmi-1. *Columns*, mean of triplicate determinations; *bars*, SD. * represents *P *< 0.05, ** represents *P *< 0.01 cisplatin plus ZOL versus cisplatin alone, Student's *t *test. Experiments were repeated at least three times and a representative experiment is shown.

Most chemotherapies for NPC are cisplatin-based combinations [23]; therefore, we explored whether ZOL could enhance the cytotoxic activity of cisplatin. Considering that the peak serum concentrations of ZOL following 4 mg standard dose is approximately 1-2 μM before localization to the bone [24], 2 μM ZOL was used in combination with cisplatin at various concentrations. We assessed possible favorable cytotoxic interactions between two drugs in five cell lines by the MTT assay. The dose-response curves and the mean IC50 values are shown in Fig. 5A-E. In HONE1 and 6-10B cell lines, which showed high expression of CENP-F, the dose-response curves of cisplatin were clearly shifted to left by ZOL, and the IC50 changed over 2-fold in combination with ZOL (from 8.10 ± 1.61 μM, 4.03 ± 0.74 μM to 3.93 ± 0.76 μM, 1.88 ± 0.39 μM, respectively) (Fig. 5A and 5B). In CNE1 and SUNE1 cell lines, which showed medium expression of CENP-F, the dose-response curves of cisplatin were slightly shifted to left by ZOL, and the IC50 changed less than 2-fold in combination with ZOL (from 4.31 ± 1.01 μM, 3.27 ± 0.60 μM to 3.44 ± 0.87 μM, 2.47 ± 0.43 μM, respectively) (Fig. 5C and 5D). However, in NPEC2 Bmi-1 cells, which showed low expression of CENP-F, the dose-response curve of cisplatin was not shifted by ZOL, and the IC50 was not changed obviously by combination with ZOL (from 7.37 ± 1.25 μM to 6.82 ± 1.49 μM) (Fig. 5E). To avoid masking the enhanced cytotoxic effect, we then selected the concentration of cisplatin to obtain an approximately 30% inhibition of proliferation *in vitro*. Analysis demonstrated that the cytotoxic effect with addition of ZOL was different from cisplatin alone in four NPC cell lines (for HONE1 and 6-10B, *P *< 0.01; for CNE1 and SUNE1, *P *< 0.05), but not in immortalized cell lines (*P *> 0.05) (Fig. 5F).

We then evaluated the potential synergistic effects of the combination of ZOL and cisplatin by the Zheng-Jun Jin method. In HONE1 and 6-10B cells, synergy was seen after treatment with 2 μM ZOL (Q = 1.54 and Q = 1.30, respectively). In CNE1 and SUNE1 cells, additivity was seen after treatment with 2 μM ZOL (Q = 0.98, Q = 0.88, respectively), whereas in NPEC2 Bmi-1, only a marginal effect was observed (Q = 0.85). These results indicates that the growth inhibition activity of ZOL in combination with cisplatin in NPC cells was correlated with the expression level of CENP-F in the cells, and a synergistic interaction from combining ZOL and cisplatin was only observed in CENP-F high expression NPC cells (HONE1 and 6-10B).

### FTI-277 Synergizes with cisplatin to enhance the cytotoxicity on high CENP-F expression cells

**Figure 6:**
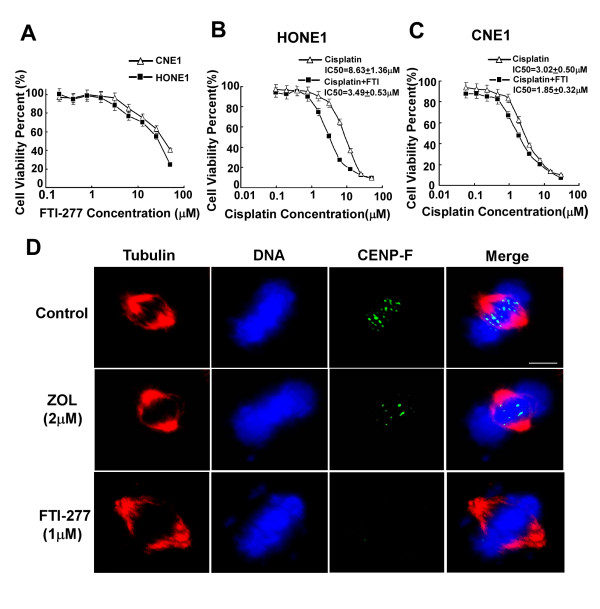
**FTI-277 Synergizes with cisplatin to enhance the cytotoxicity on high CENP-F expression cells**. A. Dose-response curves for FTI-277. The viability of each cell line (CNE1 cell line (triangle), HONE1 cell line (black square)) was plotted against the concentration of drug used in a 72 h treatment. B-C. Shifting of dose-response curves of cisplatin by ZOL. HONE1 (B) and CNE1 (C) were assayed using MTT assay. The dose-response curves of cisplatin in the presence of ZOL (black square) or the absence of ZOL (triangle). The IC50 values are shown. D. ZOL or FTI-277 reduces CENP-F from the kinetochore during metaphase and presents misaligned chromosomes. Immunofluorescence staining of Tubulin, DNA and CENP-F in untreated and drug treated HONE1 cells. Representative mitotic cells in metaphase are shown for control and drug treated cells. *Bar*, 5 μm. Experiments were repeated at least three times and a representative experiment is shown.

To determine whether these observations could be extended to other agents affecting CENP-F functionality, we did similar experiments in CNE1 and HONE1 with the commercially-available chemical FTI-277, which targets farnesyl transferase. The dose-response curves of FTI-277 are shown in Fig. 6A. We found that the dose-response curves of cisplatin in combination with 1 μM FTI-277 were clearly shifted to left (Fig. 6B), and the Q value was 1.343 in HONE1 cells, whereas, the dose-response curves of cisplatin were slightly shifted to left by FTI-277 (Fig. 6C) and the Q value was 1.075 in CNE1 cells. We also used the immunofluorescence analysis to assess the effect of ZOL or FTI-277 on CENP-F expression and chromosome localization in HONE1 cells. As shown in Fig. 6D, CENP-F was apparently reduced in metaphase, and the misaligned chromosome was observed, which might correlate with the reduced CENP-F. Therefore, we concluded that FTI-277 enhanced the cytotoxic effect of cisplatin in high CENP-F expression NPC cells, which correlated with inhibition of CENP-F in the cells.

## Discussion

In this study, we revealed that CENP-F is upregulated in NPC cell lines and NPC specimens at both the mRNA and protein levels in comparison with noncancerous nasopharyngeal epithelial cells and tissues. Overexpression of CENP-F was significantly associated with advanced clinical stage, higher T classification, skull-base invasion, and distant metastasis. Moreover, as an independent prognostic factor, overexpression of CENP-F was inversely correlated with the prognosis of NPC patients. Additionally, we found that ZOL or FTI-277 could significantly enhance the chemosensitivity to cisplatin of NPC cell lines with high expression of CENP-F, but not in cell lines with low expression of CENP-F, suggesting that CENP-F is a potential target of ZOL or FTI-277 and expression of CENP-F has potential therapeutic implications in NPC chemotherapy.

Our study suggests that CENP-F plays an important role in the progression of NPC. Upregulation of CENP-F was identified at both the transcriptional and translational levels in NPC cell lines in comparison with a primary NPEC2 and an immortalized NPEC2 Bmi-1 cells. In addition, high levels of CENP-F were detected in approximately half (48.5%) of NPC lesions. The importance of CENP-F in the progression of NPC was further highlighted by our finding that it is correlated with advanced stages and T classification, which was in general agreement with other tumor types [17,20,25,26]. Importantly, the current study was the first to identify an inverse correlation of CENP-F with skull-base invasion and distant metastasis, strongly suggesting that CENP-F could be used as a valuable factor to identify subsets of NPC patients with more aggressive tumors. Finally, our data show that the high expression of CENP-F correlates with poor prognosis and that the level of CENP-F is a potential independent prognostic factor for NPC, suggesting a function of CENP-F upregulation in the multistage pathogenesis of this disease. These findings are consistent with a previous study on breast cancer [20].

A large amount of data collected from human tumors suggests that chromosome instability (CIN) plays a causative role in a substantial proportion of malignancies and correlates with tumor grade and prognosis [27,28]. CIN, which arises as a result of an abnormal mitosis, can occur because of alterations in mitotic timing, mitotic checkpoint control, or of microtubule or centrosome dynamics [29]. CIN is commonly found in NPC and is thought to play a contributory role in tumor initiation and progression [30]. Many kinetochore proteins are proved to be associated with CIN. CENP-E is required for efficient capture and attachment of spindle microtubules and responsible for mitotic checkpoint signal transduction [31,32]. Evidence has shown CENP-E silencing leads CIN [33]. Recent research has revealed that CENP-H was upregulated in primary human colorectal cancers, and ectopic overexpression of CENP-H correlated with chromosome missegregation and aneuploidy [34]. We have previously shown that CENP-H was also upregulated in most NPC tissues [35]. The overexpression of CENP-F could affect other centromere-kinetochore components and disrupt normal kinetochore function, consequently causing mitotic delay and lagging chromosomes. Those might contribute to chromosome instability and induce the progression of NPC. A study on primary breast cancer showed that CENP-F expression was associated with CIN, including cyclin E overexpression, nuclear expression of survivin, c-*Myc *amplification, aneuploid, and high telomerase activity and poor prognosis [20]. Other studies in CENP-F depleted U2OS cells showed chromosome alignment defects, but the cells still proceeded through mitosis and became aneuploid [7]. These results suggested that the relationship between kinetochore proteins may be crucial for appropriate localization and proper functioning of the kinetochore. Our study has also provided new insight into CIN in NPC. However, further studies are needed to clarify the possible link between the biological function of CENP-F and chromosome instability in NPC, which will provide important mechanistic understanding of the role of CENP-F in the development and progression of NPC.

In addition to serving as a potential prognostic biomarker, our *in vitro *findings suggest that CENP-F may have a therapeutic implication. Numerous studies *in vitro *have shown that ZOL exerts a direct cytotoxic effect on tumor cells via inhibition of cell growth and induction of cell apoptosis, in addition to its effect on osteoclasts [36,37]. A recent study on breast cancer identified CENP-F as a potential new molecular target for ZOL, which can cause the loss of CENP-F from the kinetochore by inhibiting farnesylation and be involved in the antitumor effect by impairing correct chromosome separation [14]. Interestingly, our results did not show a direct antitumor effect of ZOL alone even at concentrations of 50 μM, which were not achievable *in vivo*, either in immortalized NPEC2 Bmi-1 or in NPC cell lines. Those data indicate that ZOL alone cannot suppress the proliferation of NPC cells at clinically relevant concentrations. However, a synergistic effect was observed when cells were treated with cisplatin in combination with a clinically relevant concentration of ZOL in high CENP-F expression NPC cells. An additive effect was observed in medium CENP-F expression cells and only a marginal effect was observed in low CENP-F expressing immortalized cells. Likewise, the combination of FTI-277 with cisplatin has been shown to have similar synergistic effects against high CENP-F expression cells. These results suggest that the effects of combined treatments are correlated with high CENP-F expression. Moreover, we found ZOL or FTI-277 causes the reduction of CENP-F from the kinetochore, which is consistent with other reports in human breast cancer cells [14] and head and neck tumor samples [13]. In addition, these results indicate that the inhibition of CENP-F might be involved in the synergistic interaction. However, the molecular mechanism underlying the synergistic effects in high CENP-F expression cells is not known, and further work is required in order to confirm this effect. Both CENP-E and CENP-F are found on the kinetochores alongside microtubules and specifically localize to the outer kinetochore plate during M-phase. A recent report has identified that an allosteric inhibitor of CENP-E motor activity can decrease CENP-E function and induce tumor cell apoptosis and tumor regression [38]. Thus, it will be important to identify novel chemicals, which target CENP-F more specifically, in order to understanding of the role of CENP-F in the development and progression of NPC as well as for the development of a novel targeted therapy.

## Conclusion

We found that CENP-F is overexpressed in NPC tissues and is positively correlated with the malignant status of NPC. High CENP-F expression is a significant prognostic factor indicating poor survival in NPC patients. Our *in vitro *findings suggest that combining cisplatin and ZOL has a synergistic effect only in high CENP-F expression NPC cells. CENP-F expression status may have important therapeutic implications for combination treatment and may be useful to stratify patients to decide on an effective strategy for anticancer therapy.

## Methods

### Patients

Freshly frozen tissue samples of twelve nasopharyngeal carcinoma biopsies and eleven noncancerous nasopharyngeal biopsies were obtained under fiberoptic nasopharyngoscopy from the Department of Nasopharyngeal Carcinoma, Sun Yat-sen University Cancer Center. A total of 202 paraffin-embedded NPC samples, which were histologically and clinically diagnosed between 1996 and 1999 at the Cancer Center, Sun Yat-sen University, were also included in this study. Patients' consent and approval from the Institute Research Ethics Committee was obtained prior to the use of these clinical materials for research purposes. The clinical characteristics of the NPC patients are described in detail in Table 4. There were 151 men and 51 women, with a median age of 48 years (ranging from 13 to 74 years). The routine staging workup included a detailed clinical examination of the head and neck, fiberoptic nasopharyngoscopy, computed tomography (CT) imaging of the entire neck from the base of the skull, chest radiography, abdominal sonography, a complete blood count, and a biochemical profile. All patients' disease stages were classified, or reclassified, according to the 1992 NPC staging system of China as described previously [2]. Clinical follow-up information was obtained from the patients' records.

**Table 4:** Clinicopathologic characteristics of 202 patients and expression of CENP-F

Factors	N (%)
Gender	
Male	151 (74.8)
Female	51 (25.2)
Age (y)	
≤ 45	90 (44.6)
> 45	112 (55.4)
Histologic classification (WHO)	
Type II	10 (5.0)
Type III	192 (95.0)
Clinical stage (92 stage)	
I	27 (13.4)
II	59 (29.2)
III	49(24.2)
IV	67 (33.2)
T classification	
T1	83 (41.1)
T2	41 (20.3)
T3	40 (19.8)
T4	38(18.8)
N classification	
N0	118 (58.4)
N1	33 (16.3)
N2	29 (14.4)
N3	22 (10.9)
Distant metastases	
No	177 (87.6)
Yes	25 (12.4)
Living status (at last follow-up)	
Alive	114 (56.4)
Death from NPC	88(43.6)
Skull-base invasion	
No	159(78.7)
Yes	43 (21.3)
Expression of CENP-F	
Low	104 (51.5)
High	98 (48.5)

### Chemicals

Zoledronic acid (ZOL) was supplied by Novartis Pharma AG (Stein, Switzerland). ZOL was dissolved in 0.9% NaCl solution as a 5 mM stock solution and stored at -20°C. FTI-277 was purchased from Sigma Chemical Co (St. Louis, MO, USA). The chemical was dissolved in DMSO at a concentration of 10 mM, and aliquots were stored at -20°C. The chemicals were diluted in fresh media before each experiment.

### Cell culture

Primary NPEC2 cultures and immortalized NPEC2 induced with Bmi-1 were established as described previously [39], and grown in keratinocyte/serum-free medium (Invitrogen, Carlsbad, CA). The NPC cell lines CNE1, CNE2, HNE1, HONE1, SUNE-1, 6-10B and C666 were maintained in RPMI 1640 (Invitrogen, Carlsbad, CA) supplemented with 10% fetal bovine serum (FBS; Hyclone, Logan, UT), penicillin (100 units/ml), and streptomycin (100 units/ml) at 37°C in a humidified 5% CO_2 _incubator.

### Real-time RT-PCR

Total RNA from different cell lines and human tissues were extracted using Trizol reagent (Invitrogen, Carlsbad, CA). After reverse transcription of the total RNA, the first-strand cDNA was then used as a template for detecting of CENP-F expression. Real-time PCR and data collection were performed with an ABI PRISM 7900HT sequence detection system. The housekeeping gene GAPDH was used as an internal control to normalize the expression levels of CENP-F. The primer sequences are sense 5'- GTAGAGGACCAACACCTGCTACC-3', antisense 5'-GTCAGCAAACCCTTTCTTTACAACT-3' for CENP-F, and sense 5'-CTCCTCCTGTTCGACAGTCAGC-3', antisense 5'- CCCAATACGACCAAATCCGTT-3' for GAPDH. To ensure reproducibility of results, all genes were tested in triplicate.

### Western blot analysis

Western blot analysis was performed as described previously [39]. Briefly, cells were harvested and lysed in lysis buffer. The protein concentration was determined by the Bradford dye method (Bio-Rad Laboratories, Hercules, CA). Equal amounts of cell extract were subjected to electrophoresis in 4% SDS-PAGE and transferred to polyvinylidene difluoride membranes (Amersham Pharmacia Biotech, Piscataway, NJ). The membrane was probed with an anti-CENP-F rabbit polyclonal antibody (1:1000; Bethyl Laboratories, Montgomery, TX). Expression of CENP-F was determined with horseradish peroxidase-conjugated anti-rabbit immunoglobulin G (1:2000; Santa Cruz Biotechnology, Santa Cruz, CA) and enhanced chemiluminescence (Amersham Pharmacia Biotech, Piscataway, NJ) according to the manufacturer's suggested protocols. An anti-α-tubulin mouse monoclonal antibody (1:1000; Santa Cruz Biotechnology) was used to confirm equal loading.

### Immunohistochemical staining (IHC)

IHC staining was performed using the Dako Envision system (Dako, Carpinteria, CA) following the manufacturer's recommended protocols. All paraffin-embedded specimens were cut into 4 μm sections and baked for 1 h at 65°C. All sections were deparaffinized with xylenes and rehydrated with graded ethanol to distilled water. Sections were submerged in EDTA antigen retrieval buffer (pH 8.0) and microwaved for antigen retrieval. After being treated with 0.3% H_2_O_2 _for 15 min to block the endogenous peroxidase, the section were treated with normal goat serum for 30 min to reduce the nonspecific binding and then rabbit polyclonal anti-CENP-F antibody (1:200; Bethyl Laboratories) overnight at 4°C. After washing, the sections were incubated with biotinylated anti-rabbit secondary antibody (Zymed) followed by further incubation with streptavidin-horseradish peroxidase (Zymed) at 37°C for 30 min. For color reaction, diaminobenzidine (DAB) was used. For negative controls, the antibody was replaced by normal goat serum.

The immunohistochemically stained tissue sections were scored independently by two pathologists blinded to the clinical parameters. The final score for CENP-F was the average of the scores obtained by the two observers. Cases with major discrepancies in scoring (i.e., > 1) were reviewed by both observers on a multiheaded microscope. Based on previous studies [40,41], we used the intensity and extent of the staining to assess CENP-F. The entire tissue section was observed to assign scores. The staining intensity was scored as 0 (no staining), 1 (weak staining exhibited as light yellow), 2 (moderate staining exhibited as yellow brown), or 3 (strong staining exhibited as brown). Extent of staining was scored as 0 (0%), 1 (1 to 25%), 2 (26 to 50%), 3 (51 to 75%), or 4 (76 to 100%), according to the percentages of the positive staining areas in relation to the whole carcinoma area or entire section for the normal samples. The sum of the intensity and extent scores was used as the final staining score (0 to 7) for CENP-F. For the purpose of statistical evaluation, tumors having a final staining score of < 3 were grouped into low CENP-F expression and those with scores ≥3 were grouped into high CENP-F expression.

### In Vitro Cytotoxicity Assays

Cytotoxicity tests were evaluated by 3-(4,5-dimethylthiazol-2-yl)-2,5-diphenyltetrazolium bromide (MTT) (Sigma, St. Louis, MO, USA) assay. Cells were grown in 96-well microtiter plates at appropriate densities and allowed to adhere for 24 h before addition of ZOL or FTI-277 alone or cisplatin and ZOL or FTI-277 together. To determine the cytotoxicity of ZOL and FTI-277, cells were exposed to increasing concentrations ranging from 0.1 μM to 50 μM for 72 h. The absorbance was determined at 570 nm in a multi-detection microplate reader (SpectraMax M5). To test the effect of ZOL or FTI-277 on the chemosensitivity of immortalized NPEC and NPC cells, ZOL (2 μM) was added to the medium with various concentrations of cisplatin in NPEC2-Bmi-1, SUNE1, CNE1, HONE-1 and 6-10B, and FTI-277 (1 μM) was added in CNE1 and HONE1. The concentrations required to inhibit growth by 50% (IC50) were calculated from survival curves using the Bliss method [42]. The results from the assays were analyzed for the combination effect between ZOL and cisplatin according to the Zheng-Jun Jin method [43]. This method provides a Q value, where Q < 0.85 indicates antagonism, 0.85 ≤ Q < 1.15 indicates additivity and Q≥ 1.15 indicates synergism. The formula is Q = E_a+b_/(E_a_+E_b_-E_a_×E_b_), where E_a+b_, E_a _and E_b _are the average effects of the combination treatment, ZOL only and cisplatin only, respectively. All treatments were performed in quadruplicate and experiments were repeated three times.

### Immunofluorescence analysis

Immunofluorescence analysis was performed as described previously [39]. Cell lines were plated on culture slides (Costar, Cambridge, MA), treated with ZOL or FTI-277 for 24 h, then fixed in ice-cold acetone for 5 min at -20°C. The cells were blocked for 30 min in 10% BSA (Sigma-Aldrich St. Louis, MO) in PBS and then incubated with rabbit polyclonal anti-CENP-F antibody (1:200; Bethyl Laboratories) for 2 hours at room temperature. After three washes in PBS, the slides were incubated for 1 h in the dark with secondary goat anti-rabbit antibodies (Invitrogen, Carlsbad, CA). After three further washes, the slides were stained with 4-,6-diamidino-2-phenylindole (DAPI; Sigma-Aldrich St. Louis, MO) for 5 min to visualize the nuclei, and examined using an Olympus confocal imaging system (Olympus FV100).

### Statistical analysis

All statistical analyses were carried out using the SPSS 13.0 statistical software package. The significance of CENP-F mRNA levels and the MTT assays were determined by t-tests. Chi-square and Fisher's exact tests were used to analyze the relationship between CENP-F expression and clinicopathologic characteristics. Bivariate correlations between variables were calculated by Spearman's rank correlation coefficients. Survival curves were plotted by the Kaplan-Meier method and compared by log-rank test. Univariate and multivariate regression analyses were performed with the Cox proportional hazards regression model to analyze independent factors affecting prognosis. *P *< 0.05 was considered statistically significant.

## Competing interests

The authors declare that they have no competing interests.

## Authors' contributions

JC and LL performed experiments and were responsible for data collection, analysis, interpretation of the results, and writing the manuscript. SC, XZ, YM were responsible for conducting the data analysis in cooperation with ZL and LF. ML and HZ were responsible for reviewing and scoring the immunostaining of sections. CQ and JS provided clinical samples for performance of experiments and validation of data. MZ and YX were responsible for experimental design, analysis and interpretation. All authors have read and approved the final manuscript.

## Supplementary Material

Additional file 1 **Fig.S1 Expression of CENP-F is elevated in NPC cell lines by normalizing to the percentage of cells in G2/M**. A. Immortalized nasopharyngeal epithelial cells (NPEC2 Bmi-1) and NPC cells (CNE1, SUNE1, CNE2, 6-10B, HONE1 and C666) were stained for DNA content and analyzed by flow cytometry, *n *= 3. B. Western blot analysis of CENP-F protein in the same cell lines as described in A. Relative expression levels of CENP-F were determined from the Western blot using Image J program. C. Quantitative analysis of the relative levels of CENP-F by normalizing to the percentage of cells in G2/M. *Bars, *SD, *n *= 3. Click here for file

Additional file 2 **Fig.S2 CENP-F protein is overexpressed in NPC tissue samples by normalizing to the percentage of cells in mitosis**. H&E and immunohistochemical stained-slides were used for calculating the percentage of mitotic cells and CENP-F positive cells in high CENP-F expression group and low CENP-F expression group. A. Representative fields are shown by H&E staining of the NPC tissue samples. B-C. Representative fields are shown by immunohistochemical staining in low CENP-F expression group (B) and high CENP-F expression group (C). *Insets*, a magnified image of the cell indicated with the arrow. *Bar, *10 μm. D. Quantitative analysis of the mitotic cells in high CENP-F expression group and low CENP-F expression group. 50 cases from CENP-F high expression group (*n *= 25) and low expression group (*n *= 25) were picked, and ten independent and intact microscopic fields (×400), representing the NPC cancer nest, were analyzed for each case. The results were expressed as the mean (+SD) percentage of number cells, * represents *P *< 0.001, CENP-F high expression group versus low expression group, Student's *t *test. A-C (×400). Click here for file
