# Supplementary figures and images for: Prognostic significance and therapeutic implications of centromere protein F expression in human nasopharyngeal carcinoma
Source: Mol Cancer. 2010 Sep 9;9:237. doi: 10.1186/1476-4598-9-237 (PMC2944187; doi:10.1186/1476-4598-9-237)

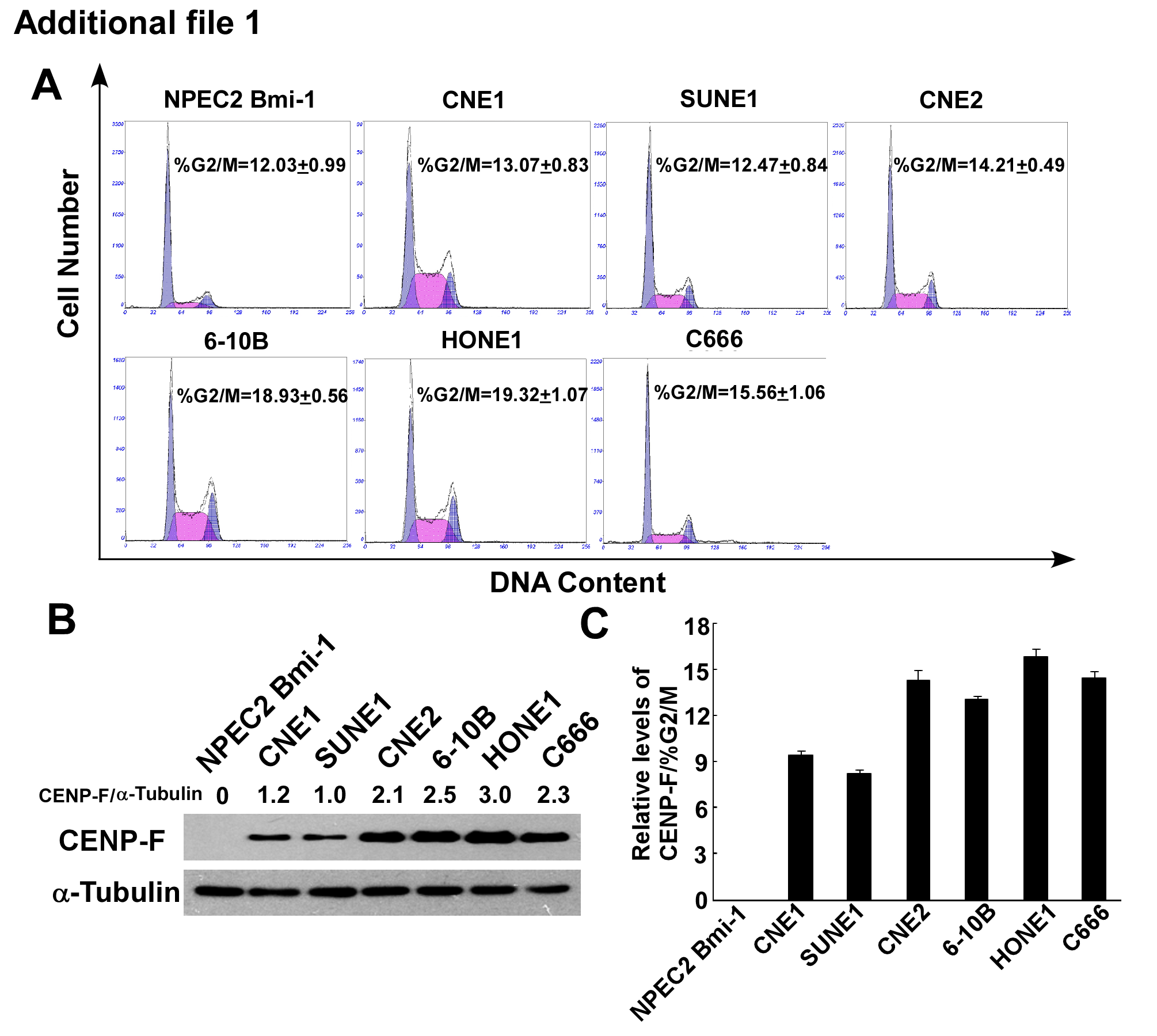

Supplement: Additional file 1 — Fig.S1 Expression of CENP-F is elevated in NPC cell lines by normalizing to the percentage of cells in G2/M. A. Immortalized nasopharyngeal epithelial cells (NPEC2 Bmi-1) and NPC cells (CNE1, SUNE1, CNE2, 6-10B, HONE1 and C666) were stained for DNA content and analyzed by flow cytometry, n = 3. B. Western blot analysis of CENP-F protein in the same cell lines as described in A. Relative expression levels of CENP-F were determined from the Western blot using Image J program. C. Quantitative analysis of the relative levels of CENP-F by normalizing to the percentage of cells in G2/M. Bars, SD, n = 3. [file 1476-4598-9-237-S1.TIFF]

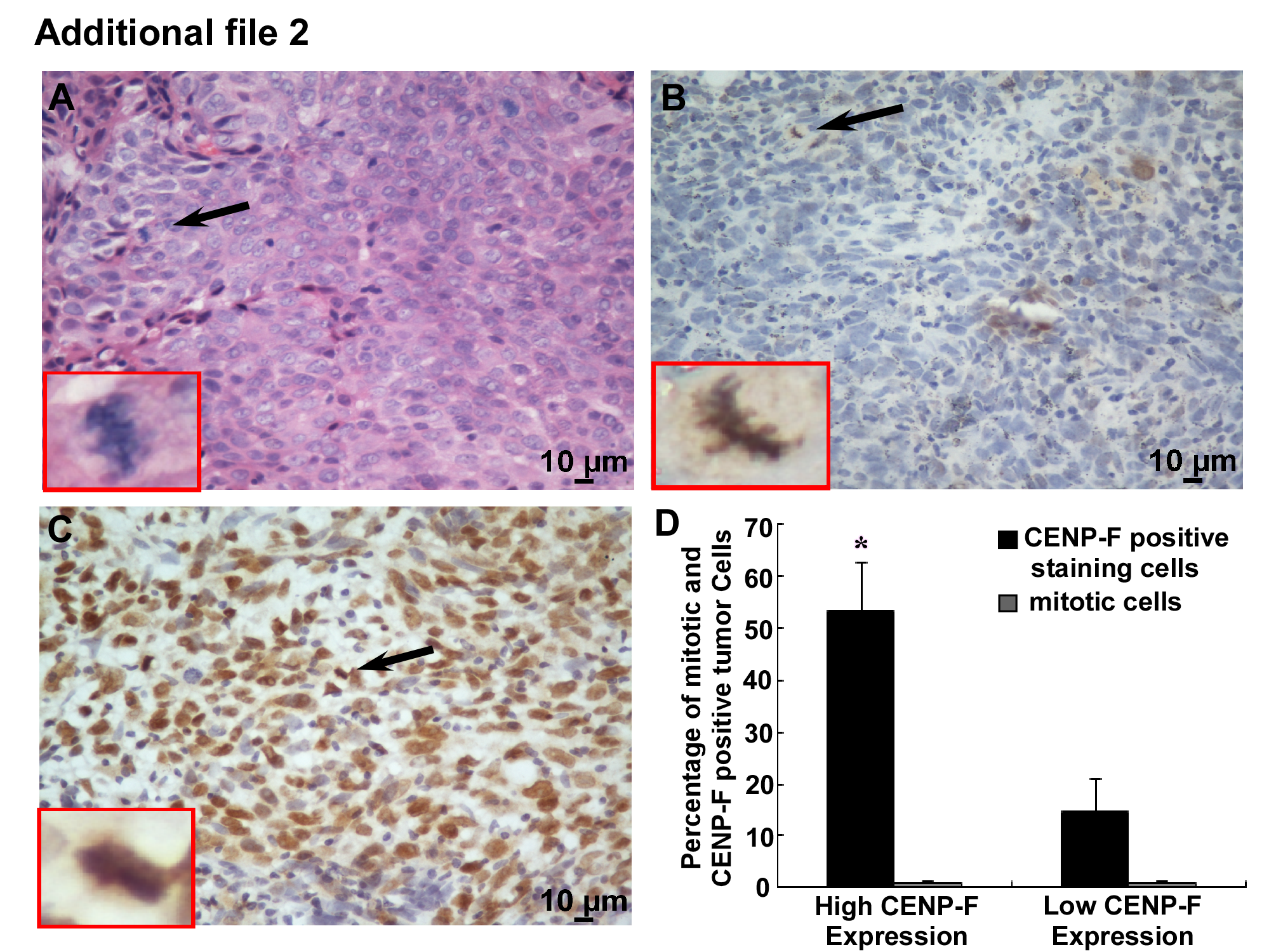

Supplement: Additional file 2 — Fig.S2 CENP-F protein is overexpressed in NPC tissue samples by normalizing to the percentage of cells in mitosis. H&E and immunohistochemical stained-slides were used for calculating the percentage of mitotic cells and CENP-F positive cells in high CENP-F expression group and low CENP-F expression group. A. Representative fields are shown by H&E staining of the NPC tissue samples. B-C. Representative fields are shown by immunohistochemical staining in low CENP-F expression group (B) and high CENP-F expression group (C). Insets, a magnified image of the cell indicated with the arrow. Bar, 10 μm. D. Quantitative analysis of the mitotic cells in high CENP-F expression group and low CENP-F expression group. 50 cases from CENP-F high expression group (n = 25) and low expression group (n = 25) were picked, and ten independent and intact microscopic fields (×400), representing the NPC cancer nest, were analyzed for each case. The results were expressed as the mean (+SD) percentage of number cells, * represents P < 0.001, CENP-F high expression group versus low expression group, Student's t test. A-C (×400). [file 1476-4598-9-237-S2.TIFF]
